# EAST MEETS WEST : APPLICATION OF AI TO SUPPORT HEALTHY AGING AND EFFECTIVE CARE IN DIVERSE CULTURES

**DOI:** 10.1093/geroni/igae098.0348

**Published:** 2024-12-31

**Authors:** Fei Sun, Guifang Guo, Yong Ding

**Affiliations:** Chair; Co-Chair; Discussant

## Abstract

This East Meets West symposium showcases five studies focused on the utilization of Artificial Intelligence (AI) in fostering healthy aging and effective care for older adults in the U.S.A. and mainland China. The first study delves into older Chinese adults’ preferences for assistive robots, highlighting their need for safety, physical functioning, and emotional support, alongside concerns regarding cost and user-friendliness. The second study introduces an innovative approach by integrating assistive robots with reminiscence music therapy to aid stroke recovery, showing promising outcomes in enhancing self-efficacy, daily living activities, positive emotions, and upper limb functionality. The subsequent three studies explore the feasibility, usefulness, and efficacy of AI-based technologies in interpreting medical records, monitoring disease progression, and providing customized health information to family caregivers. The study from the U.S. reveals that lab results interpretation generated by ChatGPT-4 demonstrated higher accuracy, relevance, and helpfulness. Similarly, the research from China is pioneering modules for early detection and comprehensive monitoring of neurodegenerative disease progression. Despite encouraging evidence, the final study points out the limitations in using GPT-3.5 Turbo for health information classification (with a focus on daily care category) into three levels with the accuracy rate falling short of expectations. In summary, these studies offer promising evidence of the benefits of AI-based innovations in promoting healthy aging and supporting caregivers in the U.S.A and mainland China. They also underline the existing limitations and adopting a person-centered approach to cater to the specific needs of older adults. After individual presentations, a discussant will provide critical feedback.

